# Identification and validation of stable reference genes for quantitative real time PCR in different minipig tissues at developmental stages

**DOI:** 10.1186/s12864-022-08830-z

**Published:** 2022-08-13

**Authors:** Jeongah Song, Jeonghee Cho, Jeongsik Park, Jeong Ho Hwang

**Affiliations:** 1 Animal Model Research Group, Korea Institute of Toxicology, Jeongeup, 56212 Republic of Korea; 2 Department of Bio-Non-Clinical Science, Graduate School of Konyang University of Bioconvergence, 158, Gwanjeodong-ro, Seo-gu, Daejeon, 35365 Republic of Korea

**Keywords:** Quantitative real time PCR, Reference genes, Minipig, RefFinder

## Abstract

**Background:**

Quantitative real time PCR (qPCR) is a powerful tool to evaluate mRNA expression level. However, reliable qPCR results require normalization with validated reference gene(s). In this study, we investigated stable reference genes in seven tissues according to four developmental stages in minipigs. Six candidate reference genes and one target gene (ACE2) were selected and qPCR was performed. BestKeeper, geNorm, NormFinder, and delta Ct method through the RefFinder web-based tool were used to evaluate the stability of candidate reference genes. To verify the selected stable genes, relative expression of ACE2 was calculated and compared with each other.

**Results:**

As a result, HPRT1 and 18S genes had lower SD value, while HMBS and GAPDH genes had higher SD value in all samples. Using statistical algorithms, HPRT1 was the most stable gene, followed by 18S, β-actin, B2M, GAPDH, and HMBS. In intestine, all candidate reference genes exhibited similar patterns of ACE2 gene expression over time, whereas in liver, lung, and kidney, gene expression pattern normalized with stable reference genes differed from those normalized with less stable genes. When normalized with the most stable genes, the expression levels of ACE2 in minipigs highly increased in intestine and kidney at PND28, which is consistent with the ACE2 expression pattern in humans.

**Conclusions:**

We suggest that HPRT1 and 18S are good choices for analyzing all these samples across the seven tissues and four developmental stages. However, this study can be a reference literature for gene expression experiments using minipig because reference gene should be validated and chosen according to experimental conditions.

**Supplementary Information:**

The online version contains supplementary material available at 10.1186/s12864-022-08830-z.

## Background

Quantitative real time-polymerase chain reaction (qPCR) is a powerful tool to evaluate mRNA expression level by measuring the fluorescent dyes binding to the amplified double-stranded DNA [1]. This technique is a useful and accurate technique due to its high sensitivity and reproductivity [2]. In addition, qPCR enables high-throughput analysis with ease. However, it needs to control for errors during experiment, such as RNA quality, initial sample amount, differences in transcriptional activity of samples, and PCR efficiency [2]. To control this error, a similar amount of good-quality RNA should be used for reverse transcription, and the gene expression level should be normalized to validated reference genes. Housekeeping genes are commonly used as a reference [2, 3]. Housekeeping genes are genes that are essential for the maintenance of cellular function, such as cell metabolism and structural gene [4]. They are expected to be expressed at a constant level across all tissue types, regardless of developmental status and experimental conditions [4]. However, this ideal housekeeping gene has not been found [5, 6]. Well-known housekeeping genes are not constantly expressed in all tissues, which might be partly because housekeeping genes have functions other than the maintenance of cellular function [7–10]. A myriad of housekeeping genes, such as glyceraldehyde-3-phosphate dehydrogenase (GAPDH), tubulins, actins, albumins, cyclophilin, microglobulins, ribosomal units (18S or 28S rRNA), ubiquitin (UBQ), and new candidate normalization genes, which were suggested using microarray [5, 11, 12], have been described. Many researchers recommended that when performing quantitative gene expression study, reference genes need to be validated to ensure stable expression in each experimental condition [13].

Researchers have tried to develop various algorithms, such as BestKeeper, NormFinder, geNorm, delta Ct, and RefFinder, with which the expression variability or stability of candidate reference genes can be measured. Bestkeeper is an excel-based software tool (http://www.gene-quantification.org/bestkeeper.html) that determines gene expression stability based on the variation of Ct values and Pearson correlation coefficient (*r*), which is calculated based on the inter-gene relation of all reference pairs and the BestKeeper Index, which is calculated as the geomean of the remaining genes. Genes with standard deviation (SD) value higher than 1 are considered unstable [14]. Normfinder was developed by Anderson et al. (2004) (https://moma.dk/normfinder-software). This algorithm calculates a stability value by combining intragroup and intergroup variation for candidate reference genes [5]. geNorm calculates the average pairwise variation for a reference gene with all other genes and presents it as M value. The lowest M value represents the most stable gene expression [15]. Delta Ct method compares the relative expression of ‘pairs of reference genes’ within each sample, and sequentially eliminates the reference gene with a large deviation in delta Ct values. Through this process, the most stable reference genes can be selected [16]. Finally, RefFinder is a free web-based software that performs an analysis based on the delta Ct method, geNorm, Normfinder, and bestkeeper. Moreover, it provides comprehensive ranking order of the candidate reference genes based on the geometric mean of ranking values obtained from the aforementioned analysis [17, 18]. These algorithms have been widely used in the studies of identification of reference genes.

Minipigs (*Sus scrofa*) are useful large experimental animals, in that they share anatomical and physiological similarities with humans, such as metabolic profile, renal system, and longer lifespan. They have been developed for biomedical research purposes since the late 1940s, resulting in at least 45 breeds of minipig breeds; and since the 1980s, the use of minipigs in research has steadily increased [19, 20]. Minipigs present many advantages over other rodents, dogs, or nonhuman primates. They have a long gestation period of approximately 114 days (d) [21], while mice, rats, and dogs have a short gestation period of 19–21 d, 21–23 d, and 58–68 d, respectively [22–24]. Minipigs have relatively large litter size (5–6 pigs per litter) and their birth weight of 400–500 g makes it easy to manipulate for dosing from an early age, or for surgical intervention, or for sampling of blood [25]. Therefore, minipigs are a useful model for juvenile toxicity studies. In addition, their long gestation period, large fetal size, and fetal weight make minipig a good model for the investigation of fetal development, including the immune system, nervous system, or specific organs.

In this study, we investigated stable reference genes in minipigs. To find out the appropriate reference genes in various tissues at different developmental stages, we measured the mRNA expression of six housekeeping genes and one target gene, angiotensin-converting enzyme2 (ACE2), in the heart, lung, liver, kidneys, stomach, intestine, and spleen of fetus (gestation d 70 (GD 70) and GD85), neonates (postnatal d 1 (PND1)) and piglets. We then analyzed the expression level of ACE2 against 6 reference genes, and compared them according to the tissues and developmental stages, to confirm their suitability for gene expression study.

## Results

### Primer validation for specificity and PCR amplification efficiency

To verify the specificity of primer pairs, melting curves were examined and agarose gel electrophoresis was performed. A single peak was detected in all primer pairs, and a single band was observed on agarose gel (Supplementary Figs. 1 and 2). PCR amplification efficiencies ranged from 90.30 to 97.19% (Table 1).

**Table 1:** Amplification efficiency of standard curves in candidate reference genes and target gene

Gene symbol	Efficiency (%)^a^	Slope	*R*^2^ values
ACE2	94.12	−3.471	0.999
B2M	96.64	−3.405	1.000
β-actin	90.30	−3.579	0.999
GAPDH	95.32	−3.439	0.997
HMBS	92.59	−3.513	0.948
HPRT1	97.19	−3.391	0.999
18S	93.01	−3.502	0.998

^a^ PCR amplification efficiency is calculated by the following formula: Efficiency = (10^–1/slope^-1) × 100

### Expression levels of various reference genes

The mRNA expression of the six candidate reference genes was measured in the seven tissues of fetus (GD 70 and 85), neonates (PND1) and 4-week-old piglets (PND28). Supplementary Table 1 shows the average Ct values, minimum or maximum values of each gene. The six candidate reference genes showed Ct values ranging 13.93 to 26.09. β-actin and 18S were relatively highly expressed genes, with Ct values ranging 13.93 to 18.63 and 14.86 to 18.94, respectively. HMBS and HPRT1 were the relatively least expressed, with Ct values ranging 18.12 to 26.09 and 21.34 to 24.98, respectively.

**Fig. 1:**
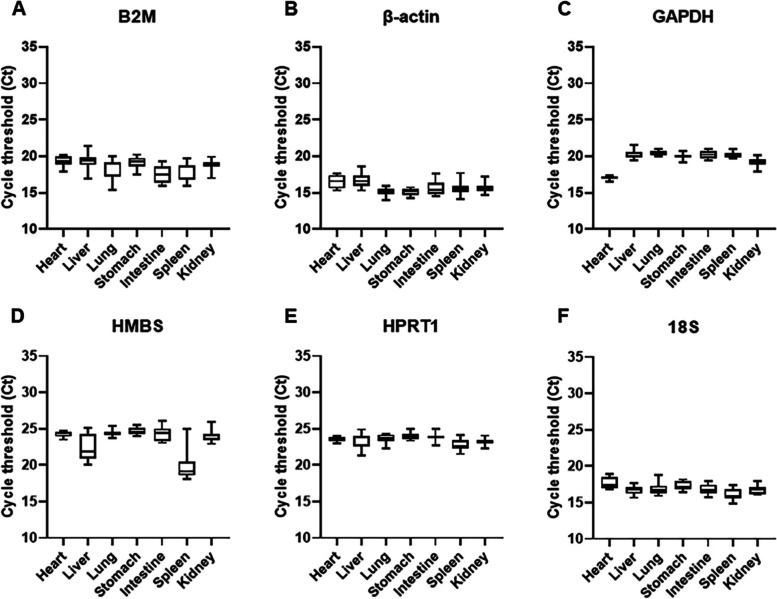
Distribution of Ct values of each reference gene across the different tissues of minipig. The expression levels are shown as median (middle line), 25th to 75th percentiles (boxes), and range (whiskers) for 15 animals of GD70 (*n* = 4), GD85 (*n* = 5), PND1 (*n* = 3), and PND28 (*n* = 3). (*n* = 15 per tissue)

Analysis of the Ct values distribution of each candidate reference gene across the different tissues showed that HPRT1 and 18S genes had lower standard deviation value, which means similar levels of HPRT1 and 18S were expressed in the seven tissues, regardless of the developmental stage (Supplementary Table 1 and Fig. 1). In contrast, HMBS and GAPDH genes had higher SD value (Supplementary Table 1 and Fig. 1). The Ct values of all six candidate reference genes were altered across the different tissues.

**Fig. 2:**
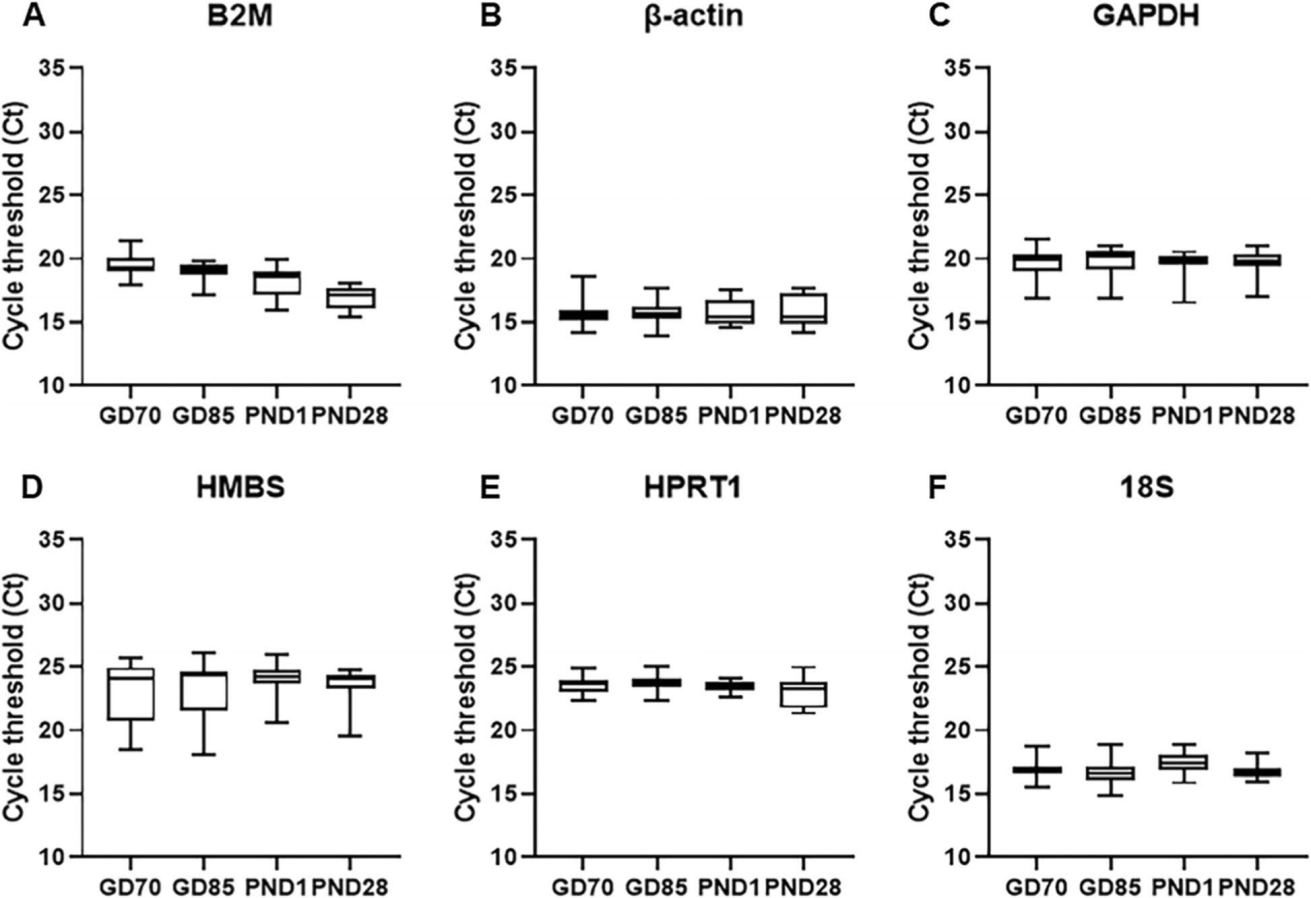
Distribution of Ct values of the reference genes at each developmental stage of minipig. The expression levels are shown as median (middle line), 25th to 75th percentiles (boxes), and range (whiskers) (*n* = 28 on GD70, *n* = 35 on GD85, *n* = 21 on PND1, and *n* = 21 on PND28)

Next, the Ct values of candidate reference genes were analyzed according to the developmental stages (Fig. 2). Four candidate reference genes except for B2M and 18S showed no differences in average Ct values, which means these four genes were stably expressed at all these developmental stages. The average Ct values significantly decreased over time in B2M gene and 18S showed a little bit higher Ct value on PND1, compared to the Ct values of other stages (Supplementary Table 1 and Fig. 2).

### Assessment of the expression stability of reference genes using web-based analysis tools

Reference genes are expected to be consistently expressed with low variation in samples. To evaluate the expression stability of candidate reference genes, statistical algorithms, BestKeeper, delta Ct, geNorm, Normfinder, and RefFinder, were employed. Using BestKeeper algorithm, the order of gene stability in all samples was as follows: HPRT1 > 18S > β-actin > GAPDH > B2M > HMBS (Table 2). The BestKeeper algorithm showed that the five candidate reference genes are considered stably expressed, excepting HMBS with SD higher than 1 [14]. The calculated stability index of delta Ct, geNorm, and Normfinder algorithms designated HPRT1 as the most stable gene, followed by 18S, β-actin, B2M, GAPDH, and HMBS (Table 3). The recommended comprehensive ranking calculated by RefFinder, which automatically calculates the geometric mean of ranking values obtained from the aforementioned these four algorithms, was also concordant with this ranking. Only BestKeeper showed swapped place of GAPDH and B2M (Table 3)

**Table 2:** Descriptive statistics and pairwise correlation of the six reference genes analyzed by BestKeeper algorithm

	B2M	b-actin	GAPDH	HMBS	HPRT	18 s
n	105	105	105	105	105	105
geo Mean [CP]	18.52	15.71	19.57	23.34	23.45	16.88
AR Mean [CP]	18.56	15.74	19.61	23.43	23.46	16.9
min [CP]	15.43	13.93	16.54	18.12	21.34	14.86
max [CP]	21.43	18.63	21.56	26.09	24.98	18.94
**std dev [+/− CP]**	**0.99**	**0.75**	**0.89**	**1.49**	**0.59**	**0.62**
CV [% CP]	5.31	4.74	4.54	6.37	2.54	3.66
min [x-fold]	−8.52	−3.43	−8.19	−37.36	−4.32	−4.07
max [x-fold]	7.51	7.53	3.97	6.73	2.9	4.15
std dev [+/− x-fold]	1.98	1.68	1.85	2.81	1.51	1.54
Pearson correlation coefficient (r)						
BestKeeper vs.	B2M	b-actin	GAPDH	HMBS	HPRT	18 s
coeff. of corr. [r]	0.536	0.479	0.054	0.698	0.625	0.479
*p*-value	0.001	0.001	0.584		0.001	0.001

*Abbreviations: n* number of samples, *geo Mean [CP]* the geometric mean of CP, *CP* crossing point, *AR Mean [CP]* the arithmetic mean of CP, *min [CP] and max [CP]* the minimum and maximum values of CP, *std. dev [+/− CP]* the standard deviation of the CP, *CV [% CP]* the coefficient of variance expressed as a percentage on the CP level, *min [x-fold] and max [x-fold]* the minimum and maximum values of expression levels expressed as an absolute x-fold over- or under-regulation coefficient, *std. dev [+/− x-fold]* standard deviation of the absolute regulation coefficients [14]

**Table 3:** Expression stability values of candidate reference genes in all samples

Gene	BestKeeper		Delta CT		Genorm		Normfinder		RefFinder	
	Rank	SD	Rank	SD	Rank	MV	Rank	SV	Rank	GM
HPRT1	1	0.59	1	1.31	1	1.02	1	0.38	1	1.00
18S	2	0.62	2	1.41	2	1.02	2	0.70	2	1.68
β-actin	3	0.75	3	1.47	3	1.11	3	0.89	3	3.00
GAPDH	4	0.89	5	1.81	5	1.38	5	1.48	5	4.73
B2M	5	0.99	4	1.61	4	1.21	4	1.17	4	4.23
HMBS	6	1.49	6	2.08	6	1.62	6	1.85	6	6.00

*Abbreviations: SD* standard deviation,* MV* M-value, *SV* stability value, *GM* geometric mean [17]

Regarding tissue type and developmental stage, Tables 4 and 5 show the comprehensive rankings. In the case of tissues, the candidate reference genes ranked top 3 were apparently variable according to tissue. GAPDH was the most stable reference gene in liver, intestine, and kidney, and the expression of HPRT1 was the most stable in spleen and stomach. B2M was the least stable gene in heart, lung, stomach, intestine, and kidneys. Regarding developmental stage, HPRT1 exhibited the most stable expression of GD70, GD85, and PND1. On PND28, the expression of 18S was the most stable. HMBS was the least stable gene, excepting PND1. Supplementary Tables 2–5 show the detailed stability index and ranking order according to the integrative RefFinder and BestKeeper, Normfinder, CT values, and geNorm.

**Table 4:** Expression stability values of candidate reference genes according to tissue type

Rank	Heart	Liver	Lung	Stomach	Intestine	Spleen	Kidney
1	HMBS	GAPDH	HMBS	HPRT1	GAPDH	HPRT1	GAPDH
2	HPRT1	18S	β-actin	GAPDH	β-actin	β-actin	HPRT1
3	GAPDH	HPRT1	HPRT1	β-actin	HMBS	GAPDH	β-actin
4	18S	B2M	GAPDH	HMBS	HPRT1	B2M	HMBS
5	β-actin	β-actin	18S	18S	18S	18S	18S
6	B2M	HMBS	B2M	B2M	B2M	HMBS	B2M

**Table 5:** Expression stability values of candidate reference genes according to developmental stage

Rank	GD70	GD85	PND1	PND28
1	HPRT1	HPRT1	HPRT1	18S
2	B2M	B2M	HMBS	B2M
3	18S	18S	B2M	β-actin
4	β-actin	β-actin	β-actin	HPRT1
5	GAPDH	GAPDH	18S	GAPDH
6	HMBS	HMBS	GAPDH	HMBS

### Assessment of the expression levels of target gene against various reference genes

The ranking order of reference genes based on the comprehensive rankings of RefFinder algorithm in all samples was HPRT1 > 18S > β-actin > B2M > GAPDH > HMBS (Table 3). To validate the stability of the candidate reference genes, we measured and analyzed the mRNA expression levels of ACE2 according to tissue type and developmental stage. Relative quantification of ACE-2 genes was performed using the 2^-ΔΔCt^ method.

**Fig. 3:**
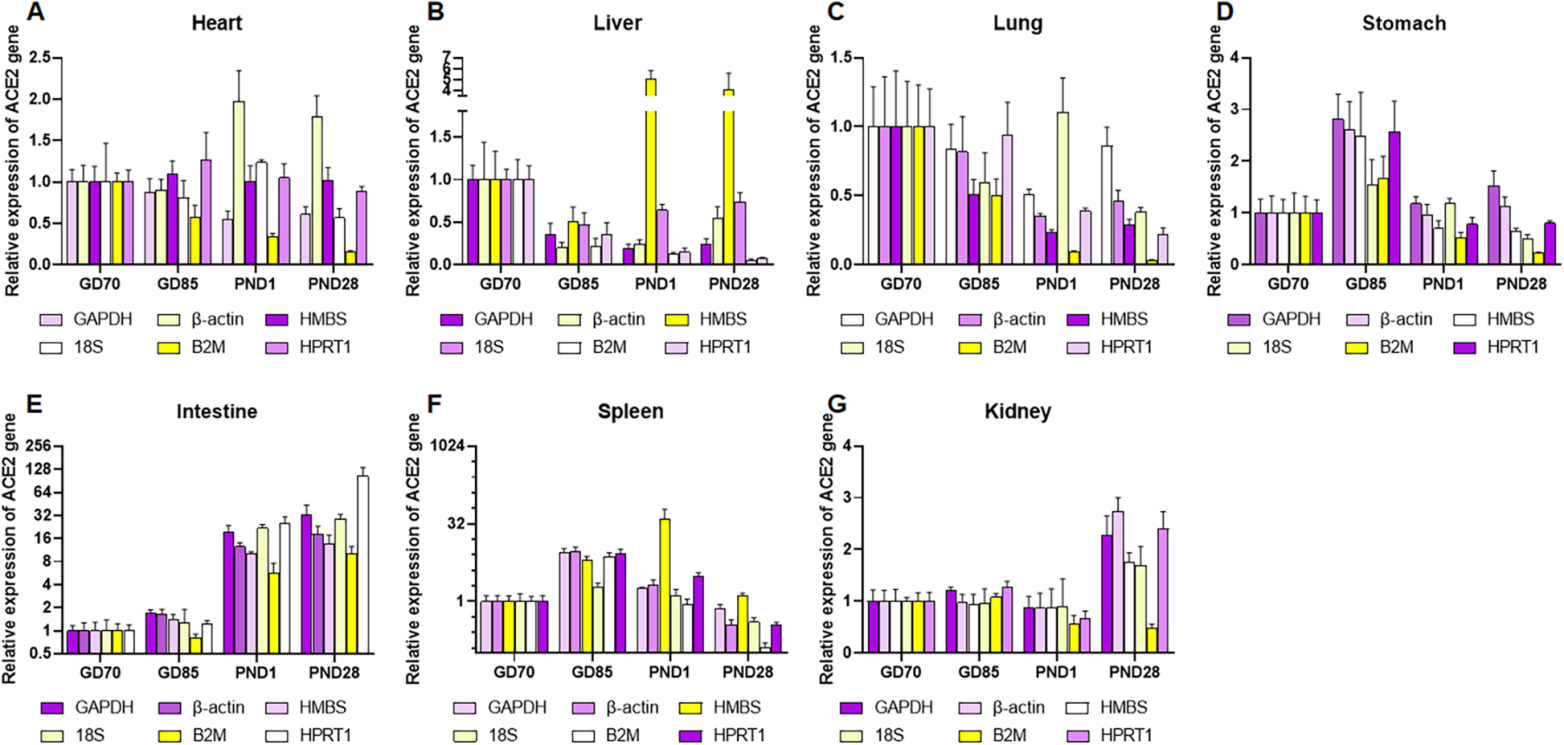
Relative expression of ACE2 normalized with each of the six reference genes across different tissues. Color of bar indicates rank of stability of the reference genes (from most stable to least stable):

**Fig. 4:**
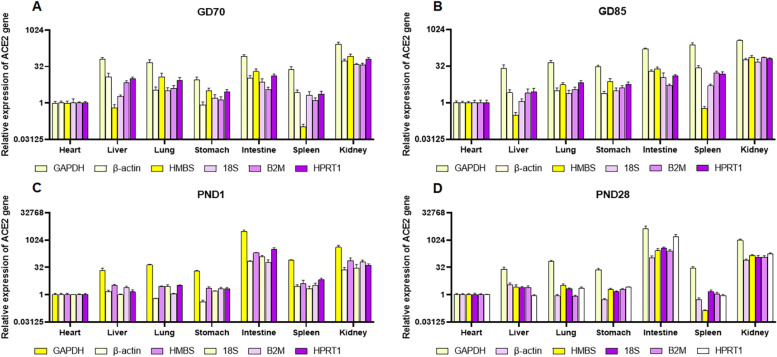
Relative expression of ACE2 normalized with each of the six reference genes at each developmental stage. Color of bar indicates rank of stability of the reference genes (from most stable to least stable):

First, the relative expression levels of ACE2 were analyzed using the candidate reference genes according to tissue type and developmental stage. ACE gene was normalized with each of six candidate reference genes. Figures 3 and 4 show that ACE2 revealed a different degree of expression change according to the reference gene used for normalization. In intestine, all candidate reference genes exhibited significant increase of ACE2 gene expression over time, though the degree of change differed among reference genes. However, in heart, liver, lung, and kidney, gene expression level using stable reference genes (HMBS, HPRT1, and GAPDH in heart; GAPDH, 18S, and HPRT1 in liver; HMBS, β-actin, and HPRT1 in lung; and GAPDH, HPRT1, and β-actin in kidney) differed significantly from those using less stable reference genes (B2M and β-actin in heart; HMBS in liver; 18S in lung; and B2M in kidney) (Figs. 3 and 4). In the case of kidney, the gene expression level of ACE2 normalized with the least stable reference gene (B2M) was 0.47 at PND28, while the relative quantity of ACE2 normalized with the most stable reference gene (GAPDH) was 2.28. When HMBS, the least stable gene in the liver, was used as reference gene, the expression of ACE2 increased at PND1 and 28, whereas the expression levels normalized with stable reference genes, GAPDH and HPRT1, significantly decreased. Generally, normalization with the three most stable genes showed similar expression pattern to ACE2.

**Fig. 5:**
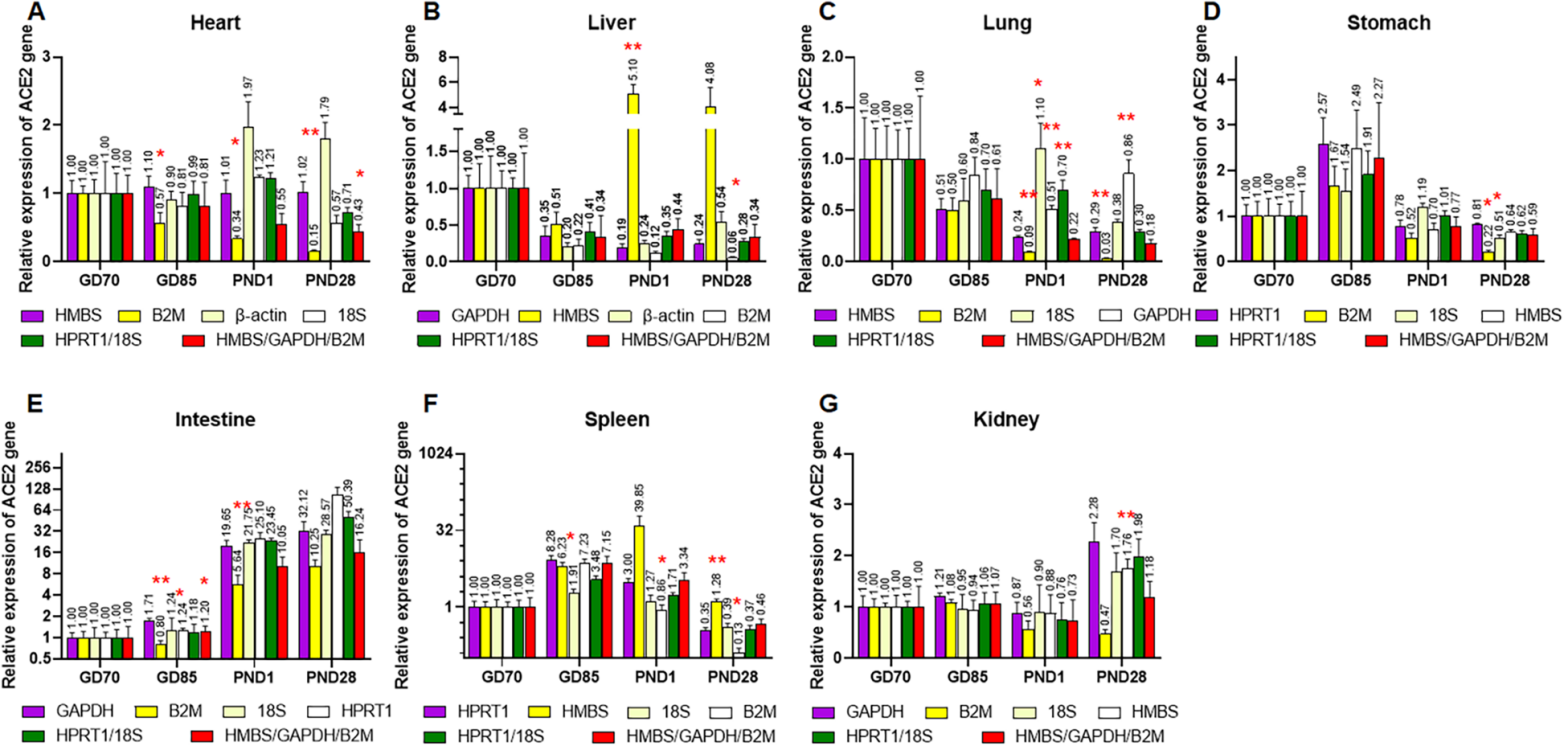
Analysis of the expression levels normalized with the most stable gene (purple box), the three least stable genes (yellow, light yellow, and white), and the geomean of HPRT1/18S (green box) and HMBS/GAPDH/B2M (red box) across different tissues. Data are expressed as mean ± SEM; * *p* < 0.05, and **p < 0.01 indicate significant difference compared to the relative expression normalized with the most stable gene of each tissue or developmental stage

**Fig. 6:**
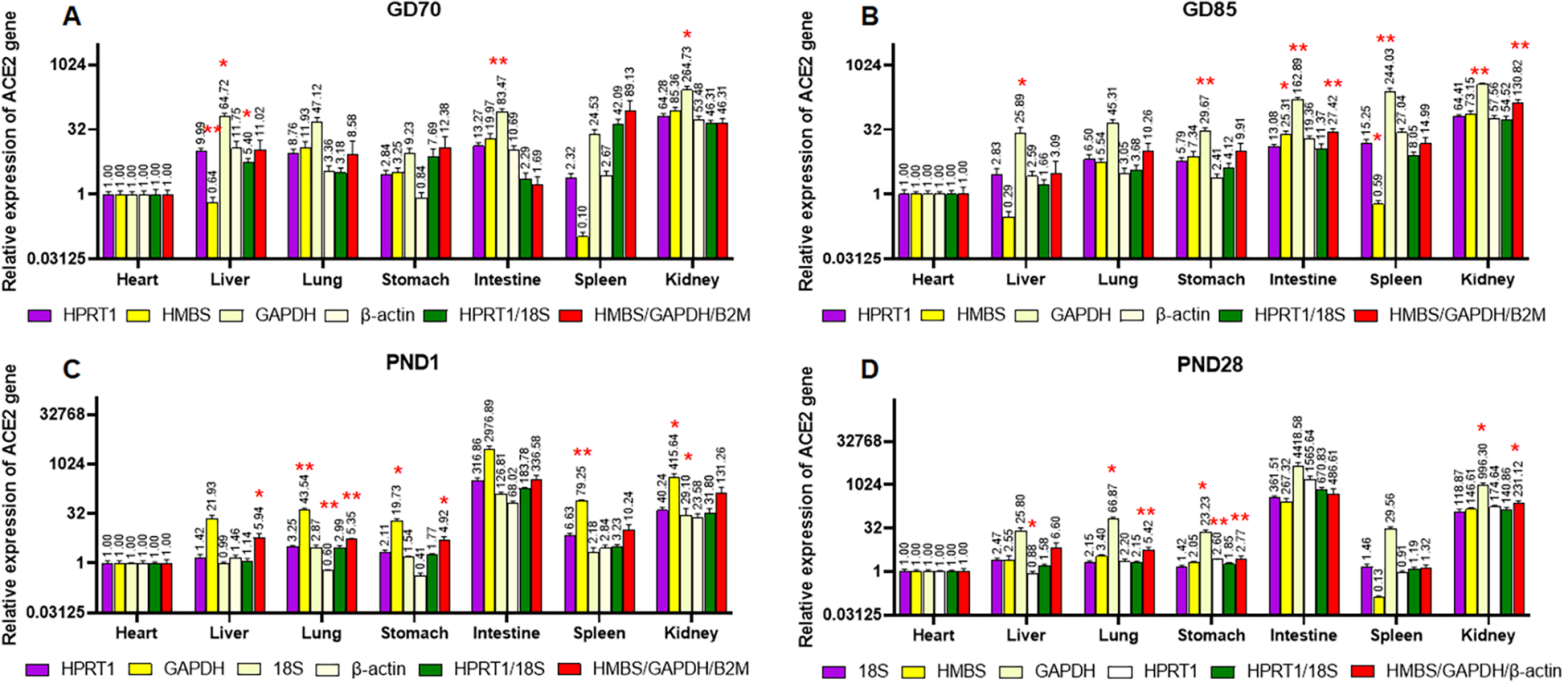
Analysis of the expression pattern normalized with the most stable gene (purple box), the three least stable genes (yellow, light yellow, and white), and the geomean of HPRT1/18S (green box) and HMBS/GAPDH/B2M (red box) at each developmental stage. Data are expressed as mean ± SEM; * *p* < 0.05, and **p < 0.01 indicate significant difference compared to the relative expression normalized with the most stable gene of each tissue or developmental stage

Next, we analyzed the expression levels normalized with multiple reference genes. Two top-ranked genes (HPRT1/18S) and three low-ranked genes (HMBS/GAPDH/B2M) of all samples were chosen. Gene expression of ACE2 were normalized with their geomean values. Figures 5 and 6 show the results. Generally, normalization with two stable candidate reference genes led to a similar expression value to that with the most stable one in each tissue. Normalization with the three least stable candidate reference genes also resulted in more similar expression pattern of ACE2 to that with the most stable one, than that with normalized with each of the three least stable candidate reference genes.

## Discussion

Quantitative real time PCR is a useful tool for quantifying gene expression levels [26]. The calculation of relative quantities of genes of interest generally requires a normalization step against reference genes. Housekeeping genes are widely used as reference, because they are ideally expected to be expressed at constant levels in all tissues under variable experimental conditions [15]. However, many studies revealed that these ideal reference genes do not exist [5, 6]. Therefore, choosing suitable reference genes is very important for the quantification of gene expression.

In this study, we investigated appropriate reference genes for different tissues at developmental stages in minipigs. The minipig is a valuable experimental model for developmental research. They have a long pregnant period (114 d) and relatively large litter size [25]. In addition, the sizes of fetuses at each gestation day or neonates are relatively large enough to handle. Our previous studies showed that fetal weights at GD42 were approximately 8.8 g, and their tissues were large enough to collect for analysis. We evaluated six candidate reference genes from 7 different tissues of a total 15 pigs (including fetuses). Overall, β-actin, 18S, and B2M had lower Ct values, and HPRT1 and HMBS showed higher Ct values, which means that β-actin, 18S, and B2M were highly expressed, whereas HPRT1 and HMBS were less expressed. Nygard et al. reported that the order of the relative expression of candidate reference genes in 17 different pig tissues (from high to low) was: B2M > β-actin > GAPDH > HPRT1 > HMBS [27], while the rank of the relative expression of the reference genes in seven tissues in the current study was: β-actin >18S > B2M > GAPDH > HMBS > HPRT1 (Table 3). The difference seems to originate from the different developmental stages of different tissue samples. The former used young female siblings, while the latter used minipigs of GD70, 85, and PND1 and 28. However, it can be generally said that β-actin, 18S, and B2M are relatively high-abundance genes, while HMBS and HPRT1 are relatively low-abundance genes [27–29].

Among the six candidate reference genes, HPRT1 showed the lowest SD of Ct values, while 18S and β-actin demonstrated the 2nd and 3rd lowest SD values in all samples. Regarding different tissues, the Ct values of HPRT1 and 18S showed lesser variability in all tissues, whereas the Ct values of GAPDH and HMBS varied across tissues, because lower Ct values of GAPDH and HMBS were detected in heart and spleen, respectively, compared to other tissues. Regarding developmental stages, B2M showed high variation of Ct values, because the Ct values of B2M decreased over time. The decrease in the average Ct value for B2M (19.44 at GD70, 19.06 at GD85, 18.19 at PND1, and 16.93 at PND28) is consistent with the other results performed by Kuijk et al. [29] and Hildyard et al. [30]. Kuijk et al. showed an increase in expression levels of B2M from oocytes to blastocysts. Hildyard et al. showed that the expression levels of B2M increased with increasing gestation days. B2M, beta-2-microglobulin, is a component of major histocompatibility (MHC) class I, and is expressed in all nucleated cells. MHC class I presents peptide derived from viral proteins of infected cells to T cell receptors, and induces adaptive immunity [31]. In addition, B2M is critical for immune cell development. B2M-knockout mice showed lack of NKT cells and mature CD8 T cells [32]. Age-associated upregulation of B2M seems to be developmentally regulated as immunity increases.

To find out stable reference genes, researchers have developed some statistical algorithms or used multiple reference in their gene expression studies. RefFinder is a valuable web-based software, which automatically calculates each stability index of delta Ct, NormFinder, geNorm, and BestKeeper and provides comprehensive ranks. RefFinder designated HPRT1 as the most stable gene, followed by 18S, β-actin, B2M, GAPDH, and HMBS in all samples. The seven different tissues and four developmental stages showed different rank from the four software, while RefFinder suggested comprehensive ranks in each tissue and developmental stage by calculating the geomean of ranks derived from the four algorithms. Generally, the high ranking orders of each algorithm seemed to be similar in the same tissue or developmental stage, though there were some exceptions, such as liver and PND1 (Supplementary Table 1 or 2). Depending on the condition of the study, it is necessary to select appropriate reference genes to measure the expression levels of the target genes. For example, in analyzing the expression levels of seven different tissues at different development stages, HPRT1 and 18S were good reference genes. When expression levels are quantified at a fixed time point, HPRT1 and B2M are stably expressed across the different tissues except PND28. At PND28, 18S and B2M were good reference genes. Though the expression levels of B2M increased from fetuses to piglets, at a fixed time B2M showed constant expression levels across the seven tissues.

To validate the candidate reference genes, the expression of ACE2 was measured and analyzed according to the various tissues and developmental stages. As expected, the gene expression pattern varied against the reference genes used for normalization. In the current study, the expression patterns normalized with the three top ranked candidate reference genes seemed generally similar to each other; however, they were quite different from those normalized with the least and less stable genes. At various developmental stages, the expression patterns of ACE2 stayed constant in heart, decreased in liver, and significantly increased in kidney at PND28; whereas, ACE2 expression levels increased in heart and liver and decreased in kidney, when normalized with the least stable genes, β-actin, HMBS, and B2M, respectively. In addition, the relative quantities normalized with B2M were significantly lower than those normalized with other reference genes at PND1 and 28, because expression levels of B2M upregulated over time (Figs. 5 and 6). Relative quantification of qPCR relies on the assumption that the reference genes are stably expressed across all tested samples, regardless of cell or tissue type, age, or treatment. Therefore, normalization with unsuitable reference gene leads to biased qPCR results.

To lessen bias induced by an unsuitable reference gene, many researchers have recommended the use of two or more reference genes for qPCR study [3, 33–35]. For the developmental study in seven tissues, we chose two sets of genes, e.g., one set of HPRT1 and 18S (the two most stable genes of all samples) and another set of HMBS/GAPDH/B2M (the three least stable genes of all samples), and analyzed the expression levels of ACE2 by normalizing with the geomean of two or three genes. The expression levels of ACE2 normalized with the two most stable reference genes, and with the most stable reference gene were generally similar. The expression patterns of ACE2 normalized with the three least stable reference genes seemed, to some extent, to compensate for false results induced by one unsuitable gene. These results imply that normalization with multiple reference genes can provide more reliable qPCR results, though they are the least stable genes. In addition, the use of more than 2 reference genes gives the research an opportunity to evaluate the stability of these genes.

ACE2 is one of the metallocarboxypeptidases that cleaves Angiotensin I into angiotensin 1–9 and angiotensin II into angiotensin 1–7. It plays a key role in diabetes, renal, and cardiovascular disease by counterbalancing the ACE/renin angiotensin aldosterone system which is associated with sodium and water retention, vasoconstriction, hypertension, and accelerated thrombosis [31]. It is also a receptor for severe acute respiratory syndrome coronavirus 2. ACE2 gene is highly expressed in the kidney, heart, small intestine, and testis and is known to be expressed in all organs [36]. In this study, we analyzed the ACE2 expression levels in the seven tissues at each developmental stage. The expression levels of ACE2 in minipigs highly increased in intestine and kidney other than tissues at PND1 and PND 28, which is consistent with the ACE2 expression pattern in humans (https://www.ncbi.nlm.nih.gov/gene/59272) [37]. This result indirectly confirmed that our candidate reference genes were good enough for qPCR study.

Dozens of papers have evaluated and validated reference genes in pigs. Martino et al. [38] analyzed the stability of reference genes in cardiac tissues of the healthy and heart failure minipigs. HPRT-1, TBP, and peptidylprolyl isomerase A (PPIA) were the most stably expressed genes in the right and left atrium, while PPIA, GAPDH, and ACTB and HPRT-1, TBP, and GAPDH were the most stable genes in right and left ventricle, respectively. Wang et al. [39] reported that B2M/HMBS/HPRT1 were the most stable gene set and GAPDH, β-actin, and 18 were the least stable genes in the gastrointestinal tract during the weaning period. Piórkowska et al. [40] evaluated the stability of 8 candidate reference genes in the adipose tissue of three pig breeds. Ornithine decarboxylase antizyme 1 (OAZ1), 60S ribosomal protein L27 (RPL27), and β-actin were the most stable genes. Nygard et al. [27] showed that β-actin, RPL4, TBP and HPRT1 were expressed at a constant level across 17 different pig tissues. Erkens et al. [28] evaluated the expression stability of 10 reference genes in the porcine backfat and longissimus dorsi muscle. β-actin, TBP, and TOP2B were the most stable, while RPL13A and SDHA were the least stable. Kuijk et al. [29] analyzed transcription levels in porcine oocytes and embryos using seven reference genes such as B2M, BACT, GAPDH, H2A, PGK1, SI8, and UBC. GAPDH was the most stable gene, while B2M was the least stable gene. These results imply that there were no stable reference genes that encompass all experimental conditions, because researchers showed that stable reference genes differ with experimental condition, namely, strain, age, and tissues of pigs or experimental animals used for experiment.

## Conclusions

In this study, we validated six candidate reference genes in seven tissues of minipigs at various stages of development. It is very difficult to find a stable reference gene across various tissues at different developing time points. We suggest that HPRT1 and 18S are a good choice for analyzing all these samples across the seven tissues and developmental stages. Also, the introduction of the multiple reference genes is strongly recommended for qPCR study. However, this study can be a reference literature for gene expression experiments using minipig, because the reference gene should be validated and chosen according to experimental conditions, as gene expression differs according to strain, tissue type, and age of minipigs. Validation of reference genes and the introduction of two or more reference genes lessen the bias, and allow more reliable qPCR data/results in gene expression studies.

## Materials and methods

### Animal experiments

Pregnant Yucatan miniature pigs were purchased from Optipharm Co., Ltd. (Cheongju, Korea). Sow and piglets were housed in a loose farrowing pen in a controlled environment (temperature of 19–25 °C, relative humidity of 50 ± 10%, and air ventilation of 10–20 times per hour (h) with a 12 h/12 h light/dark cycle). Sows were euthanized by intravenous injection of thiopental sodium (0.5 g/15 kg) after anesthesia with xylazine (0.5–3 mg/kg, intramuscular) and ketamine (15 mg/kg, intramuscular). Fetuses at GD70 and GD85 were obtained by hysterectomy. The uterus was exposed through midline abdominal incision. Fetuses were rapidly removed from uterus, and then put in plastic dishes on ice. Gestation d was calculated from the day of mating. For sampling on PND1 (day of birth), neonates were removed from sow within 2–3 h after birth. For sampling on PND28, piglets were euthanized using the same method described above. After euthanasia, heart, lung, liver, kidneys, stomach, intestine, and spleen were removed and placed in RNAlater stabilization solution (Sigma Aldrich, MO, USA). All experiments were approved by the Institutional Animal Care and Use Committee of Korea Institute of Toxicology (Approval No.: 2012–0018, 2010–0307, 2008–0250). This manuscript complies with the ARRIVE guidelines [41].

### RNA extraction and cDNA synthesis

Each tissue was stored in RNAlater stabilization solution at − 20 °C until analysis. Total RNA was extracted using RNeasy Mini kit (Qiagen, Hilden, Germany), according to the manufacturer’s protocol. RNA yield and purity were assessed using a QIAxpert® system (QIAGEN, Hilden, Germany). RNA samples with A_260/280_ and A_260/230_ > 1.8 were used. Four hundred nanogram of total RNA was transcribed using GoScript™ reverse transcription System (Promega, Madison, WI, USA), according to the manufacturer’s instructions.

### Selection of reference genes and a target gene

Glyceraldehyde-3-phosphate dehydrogenase (GAPDH), β-actin, and ribosomal protein S18 (18S) are commonly used as reference genes in qPCR studies. Hypoxanthine phosphoribosyltransferase 1 (HPRT1), Hydroxymethylbilane synthase (HMBS), and β2-Microglobulin (B2M) were included in this study from the literature [4, 27, 39, 40]. Angiotensin converting enzyme 2 (ACE2) was chosen as a target gene from the literature. This gene is expressed in many tissues including kidneys, heart, gastrointestinal tract, lung, oral cavity, and liver and the expression pattern of this gene has been extensively studied in humans (https://www.ncbi.nlm.nih.gov/gene/59272) [36, 42–44]. We can compare the expression levels of ACE2 in different tissues of minipigs with those of humans, thus indirectly validating the suitability of the reference genes. The sequence of the selected reference genes and a target gene were obtained from the GenBank database (https://www.ncbi.nlm.nih.gov/genbank/).

### Validation of primers and qPCR

PCR primers were designed using NCBI BLAST (https://www.ncbi.nlm.nih.gov/tools/primer-blast/index.cgi?LINK_LOC=BlastHome) or Primer Express® software v3.0.1 (Applied Biosystems, Foster City, CA, USA). Table 6 lists the information of reference genes and primers. The specificity of the primers was checked using the melting curve and agarose gel electrophoresis. PCR amplification efficiency (E% = (10^(− 1/slope)^-1) × 100) was measured by means of standard curves. Adult spleen sample was used for this validation.

**Table 6:** Reference genes used in this study and primer

Gene symbol (gene name)	GenBank Accession number	Primer forward (F)/reverse (R)	Amplicon size
ACE2 (angiotensin converting enzyme 2)	NM_001123070.1	F: TGCCAGGGCCAAATGGT R: ATCTAGTGGATACGTTTTGGCAAT	67
B2M (beta-2-microglobulin)	NM_213978.1	F: CGGAAAGCCAAATTACCTGA R: CATCTTCTCCCCGTTTTTCA	88
β-actin (beta-actin)	XM_021086047.1	F: GGATGCAGAAGGAGATCACG R: ATCTGCTGGAAGGTGGACAG	130
GAPDH (Glyceraldehyde 3-phosphate dehydrogenase)	NM_001206359.1	F: CCACCCAGAAGACTGTGGAT R: AAGCAGGGATGATGTTCTGG	81
HMBS (hydroxymethylbilane synthase)	DQ845174.1	F: AGGATGGGCAACTCTACCTG R: GATGGTGGCCTGCATAGTCT	83
HPRT1 (hypoxanthine phosphoribosyltransferase 1)	NM_001032376.2	F: AAGCTTGCTGGTGAAAAGGA R: GTCAAGGGCATAGCCTACCA	100
18S (18S ribosomal RNA)	NR_046261.1	F: CCCACGGAATCGAGAAAGAG R: TTGACGGAAGGGCACCA	122

mRNA levels were quantified using SYBR Green on the Applied Biosystems QuantStudio 5 (Foster City, CA, USA). PCR reactions were performed in a final volume of 20 μL containing 10 μL of SYBR Green PCR Master Mix (Applied Biosystems, Woolston, Warrington, UK), 5 μM of each primer, 5 μL of diluted cDNA, and distilled water. The PCR conditions were 95 °C for 10 min, followed by 40 cycles of 15 s at 95 °C and 1 min at 60 °C. Four or five 5-fold serial dilution of a pool of cDNA samples was used to construct standard curves to evaluate the efficiency of primer pairs.

The relative expression levels of target genes were calculated using the 2-ΔΔCt method [45]. All reactions were carried out in three to five biological replicates and two technical replicates.

### Analysis of the expression stability of candidate reference genes

First, we calculated the mean value and standard deviation of the threshold cycle (Ct) value of six candidate reference genes in a total 105 samples. Then, the mean and SD were recalculated according to tissue type and developmental stage.

To evaluate the stability of the candidate reference genes, we used BestKeeper, geNorm, NormFinder, and delta Ct method through the RefFinder web-based tool (https://heartcure.com.au). This statistical analysis was also performed for all samples, according to tissue type and developmental stage. The stability of candidate reference genes was analyzed from each statistical algorithm, and then ranked in most stable to least stable order.

Next, the relative expression of ACE2 was calculated in all samples according to the different tissues and developmental stages to validate the candidate reference genes. Normalization was performed using each of six candidate reference genes, the geomean of the two most stable genes, or the geomean of the three least stable genes. Relative mRNA quantity was expressed as fold change relative to the average of GD70 or heart. Relative quantification of ACE2 genes was performed using the 2^-ΔΔCt^ method, where ΔΔCt = [(Ct_ACE2_ − Ct_reference gene_)_GDx or PNDx_ − (Ct_ACE2_ − Ct_reference gene_)_GD70_] or ΔΔCt = [(Ct_ACE2_ − Ct_reference gene_)_tissue_ − (Ct_ACE2_ − Ct_reference gene_)_heart_] [45].

### Data analysis

To determine the difference of Ct values of reference genes according to the different tissues and developmental stages, one-way analysis of variance with Dunnett’s test was carried out using SPSS 12 (SPSS, Chicago, IL, USA, 2005). The statistical significance of the relative expression of ACE2 was evaluated with Student’s t test using SPSS 12 (SPSS, Chicago, IL, USA, 2005).

## Supplementary Information

**Additional file 1.**

**Additional file 2.**

## Data Availability

All data generated or analyzed during this study are included in this published article and its supplementary information files.
