# Supplementary material for: Identification and validation of stable reference genes for quantitative real time PCR in different minipig tissues at developmental stages
Source: BMC Genomics. 2022 Aug 13;23:585. doi: 10.1186/s12864-022-08830-z (PMC9374586; doi:10.1186/s12864-022-08830-z)
Supplement: Supplementary file 1 — Additional file 1. [file 12864_2022_8830_MOESM1_ESM.docx]

Supporting information

Identification and validation of stable reference genes for quantitative real time PCR in different minipig tissues at developmental stages

Jeongah Song^1*^, Jeonghee Cho^1,2^, Jeongsik Park^1^, Jeong-Ho Hwang^1**^

^1^ Animal Model Research Group, Korea Institute of Toxicology, Jeongeup 56212, Republic of Korea

^2^ Graduate School of Konyang University of Bioconvergence, Department of Bio-Non-Clinical Science, 158, Gwanjeodong-ro, Seo-gu, Daejeon 35365, Republic of Korea

 *Corresponding author

Jeongah Song, Ph.D., D.V.M.

Animal Model Research Group, Korea Institute of Toxicology,

Jeongeup 56212, Republic of Korea.

Tel.: +82-63-850-8553

E-mail: jasong@kitox.re.kr

** Corresponding author

Jeong-Ho Hwang, Ph.D.

Animal Model Research Group, Korea Institute of Toxicology,

Jeongeup 56212, Republic of Korea

Tel.: +82-42-610-8528

e-mail: jeongho.hwang@kitox.re.kr


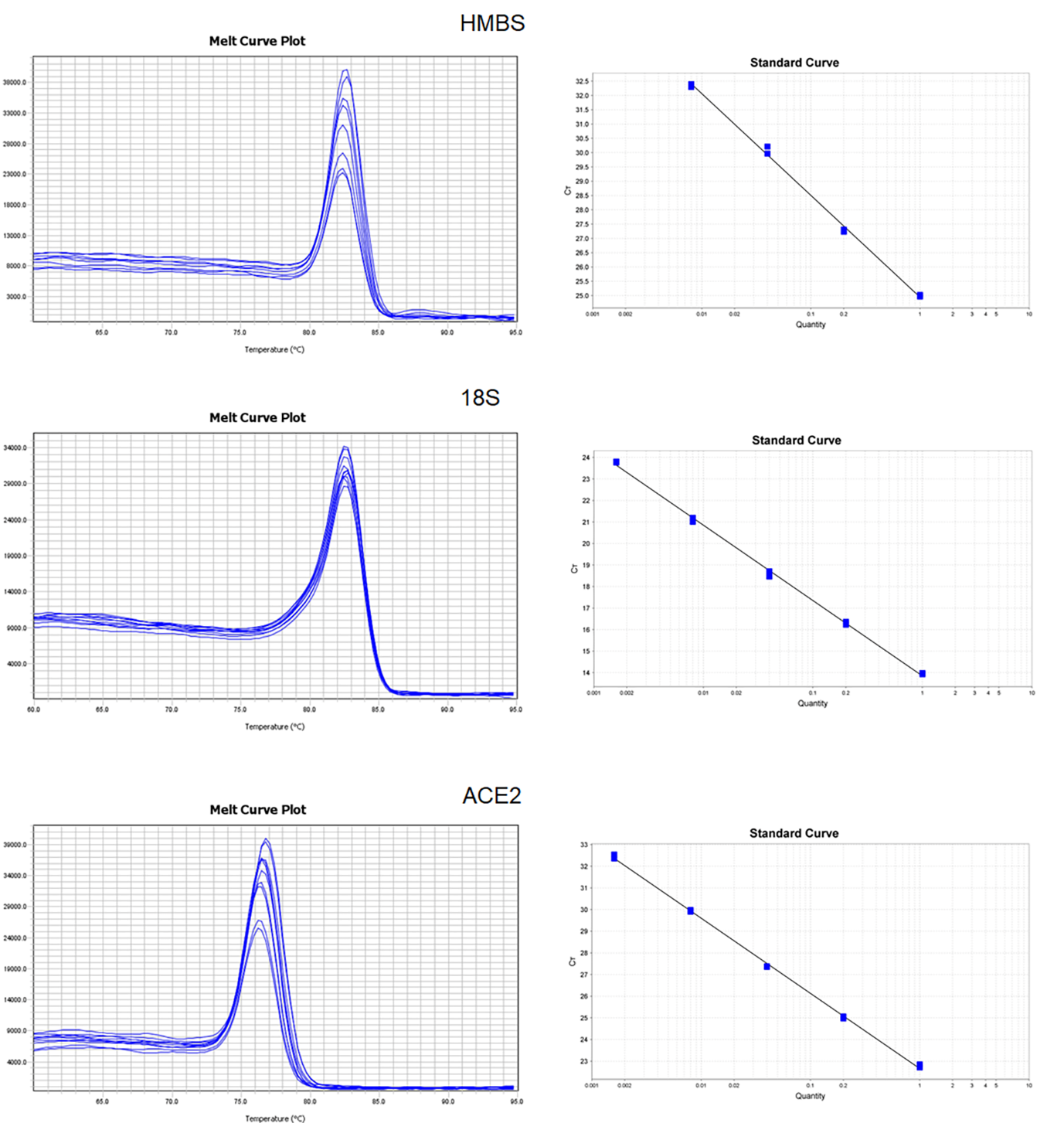

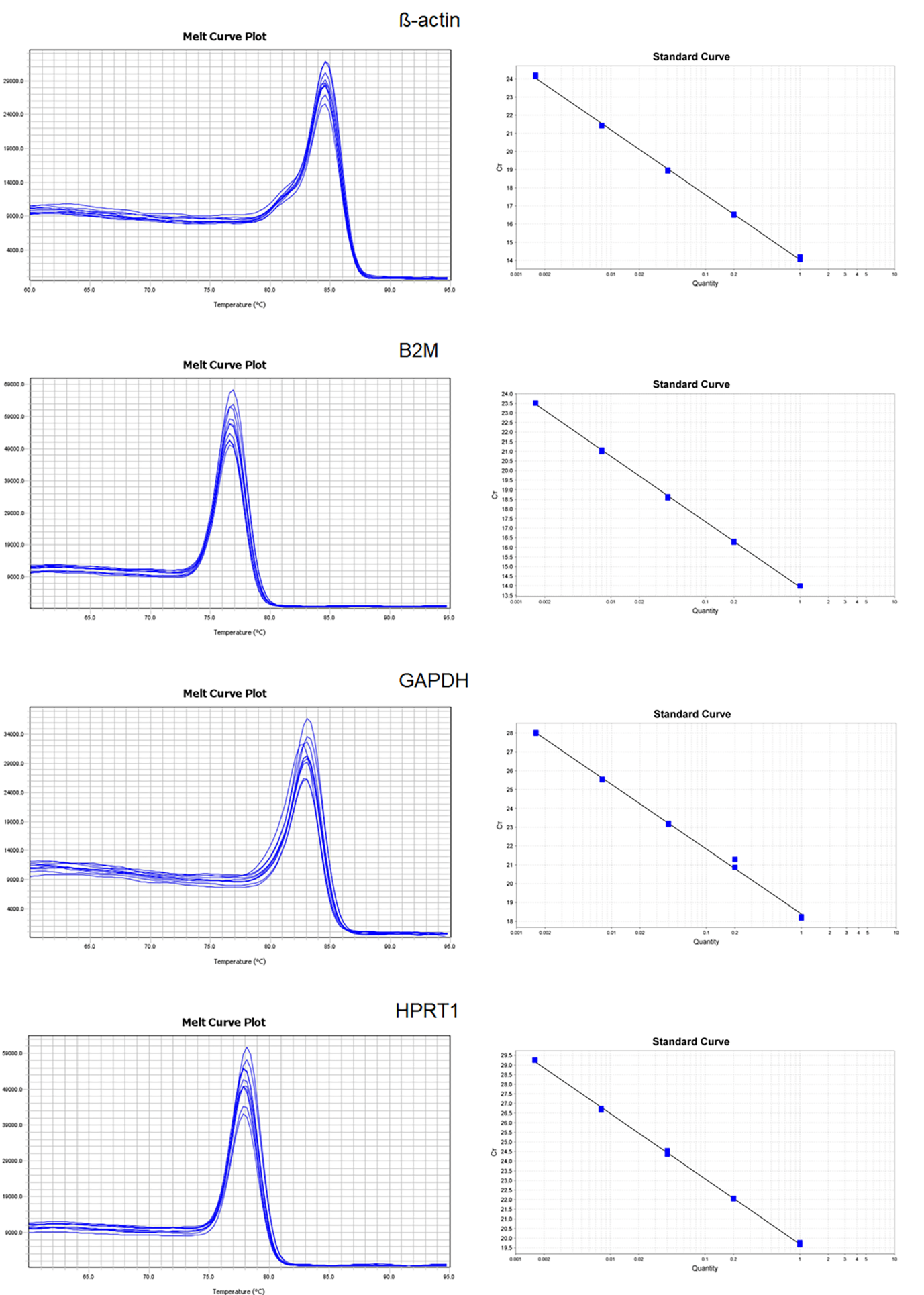


Supplementary Fig. 1 Melting curves and standard curve of the candidate reference genes.


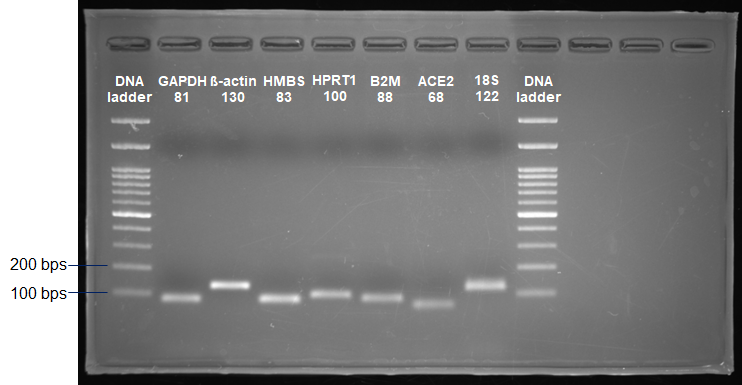


Supplementary Fig. 2 Gel electrophoresis of qPCR products. Single band of expected size by each primer pair was confirmed on a 2 % agarose gel.
